# Positron emission tomography-magnetic resonance imaging (PET-MRI) for response assessment after radiation therapy of cervical carcinoma: a pilot study

**DOI:** 10.1186/s13550-017-0352-6

**Published:** 2018-01-02

**Authors:** J. E. Mongula, F. C. H. Bakers, S. Vöö, L. Lutgens, T. van Gorp, R. F. P. M. Kruitwagen, B. F. M. Slangen

**Affiliations:** 1 Department of Obstetrics and Gynecology, GROW - School for Oncology and Developmental Biology, Maastricht University Medical Centre, Postbus 5800, 6202 Maastricht, AZ The Netherlands; 2 Department of Radiology, GROW—School for Oncology and Developmental Biology, Maastricht University Medical Centre, Maastricht, The Netherlands; 3 Department of Nuclear Medicine, GROW—School for Oncology and Developmental Biology, Maastricht University Medical Centre, Maastricht, The Netherlands; 4 Department of Radiation Oncology (MAASTRO), GROW—School for Oncology and Developmental Biology, Maastricht University Medical Centre, Maastricht, The Netherlands

**Keywords:** Cervical cancer, MRI, PET-MRI, PET, Radiation therapy

## Abstract

**Background:**

Advanced stage cervical cancer is primarily treated by radiotherapy. Local tumor control is a prerequisite for cure. Imaging after treatment is controversial. Positron emission tomography (PET) combined with computer tomography (PET-CT) shows great promise for detecting metastases. On the other hand, magnetic resonance imaging (MRI) is superior in depicting anatomical details. The combination of PET-MRI could result in more accurate evaluation of cervical cancer treatment outcome. The aim of this pilot study is to share our initial experience with PET-MRI in the evaluation of treatment response in cervical cancer after radiation treatment.

**Methods:**

Ten patients with cervical carcinoma (FIGO ≥IB2) were prospectively evaluated. Eleven weeks (median; range 8–15 weeks) after radiation therapy, treatment response was evaluated by PET-MRI. The PET, MRI, and combined PET-MRI images were evaluated for the presence of local residual tumor and metastasis. Diagnostic performance was assessed by area under the receiver operator characteristic (ROC) curve for evaluation of local residual tumor. The readers were blinded for outcome data. Local residual disease, metastasis, diagnostic confidence, and change of opinion were scored on a 5-point Likert scale. The reference standard consisted of pathology and/or follow-up according to the clinical guidelines.

**Results:**

Three out of ten patients had local residual abnormalities suggestive for tumor residue after radiation treatment. The availability of both PET and MRI resulted in an increase in diagnostic confidence in 80–90% of all patients. Change of opinion was observed in 70% and change of policy in 50%, especially in the group with residual tumor. The diagnostic accuracy increased significantly for the radiologist if PET-MRI was combined (AUC .54 versus .83).

**Conclusions:**

PET-MRI shows promise for evaluation of treatment response after radiation for cervical cancer, especially increasing diagnostic confidence, while potentially increasing diagnostic performance.

## Background

Radiation treatment for cervical cancer is recommended for advanced disease (International Federation of Gynecology and Obstetrics (FIGO) stages ≥IB2). In advanced disease, one third of the patients will develop recurrent or progressive disease, the majority occurring in the first 2 years [1]. Local persistent tumor is responsible for one fourth of treatment failure; lymph node metastasis and distant metastasis are responsible for the remaining part [2].

For the first group of patients (local persistent tumor), potential therapeutic options depend on the extent of the local tumor and/ or the presence of metastasis [1].

To date, routine surveillance imaging after radiation treatment is not used in daily practice, partly because of poor performance of diagnostic tests [3]. Second, the timing of the follow-up evaluation remains unclear as tumor regression rate varies. Early assessment could lead to an overestimation of residual tumor; on the contrary, late assessment could miss the opportunity to treat asymptomatic persistent disease [4].

Fluorodeoxyglucose-positron emission tomography (FDG-PET) has high potential for predicting response and metastasis after treatment [5–7]. Siva et al. evaluated therapy response by PET-CT 3 months after treatment [7]. Besides excellent survival for the complete response group, they also showed a 78% overall survival after salvage therapy for patients with isolated local and nodal residual disease.

In general, FDG-PET is combined with low-dose computed tomography (CT) images. The morphologic resolutions of these CT images are rather poor as compared to MRI. As a consequence, if local treatment failure is suspected based on PET-CT, additional magnetic resonance imaging (MRI) is necessary for adequate treatment planning [8]. However, the detection of persistent tumors by MRI is complicated by the occurrence of false positive results, mainly due to post-radiation-induced fibrosis, inflammation-induced edema, and necrosis. MRI criteria sets and diffusion-weighted MRI imaging could decrease false positive results to some extent but a further increase in diagnostic performance is warranted [9, 10]. The combination of the superior morphologic MRI with functional FDG-PET imaging could result in a more accurate diagnosis, staging, and follow-up of cervical cancer [11, 12]. The functional imaging of the FDG-PET could reduce false positive results of MRI by allowing for differentiation between residual tumor and radiation-induced fibrotic tissue. FDG uptake is not expected in fibrotic tissue in comparison to residual tumor. PET is able to assess (lymph node) metastasis, potentially resulting in a better treatment allocation after radiation treatment compared to MRI alone [5, 7].

The aim of our study was to share our initial experience with the PET-MRI in the evaluation of radiotherapy treatment to discriminate between patients with a complete local response and patients with local residual tumor. We evaluate the potential value of combined PET-MRI in comparison to the MRI and PET component.

## Methods

### Patients

This is a prospective cohort study of 11 consecutive women with histologically proven advanced stage primary cervical cancer (FIGO stages ≥IB2-IVB), which were referred to Maastricht University Medical Centre between July 2014 and October 2015. All patients were discussed in a multi-disciplinary team consisting of gynecologic oncologist, medical oncologist, radiation oncologist, pathologist, radiologist, and a nuclear physician. Patients allocated to radiotherapy (FIGO stages ≥IB2, according to the clinical guidelines) were included.

Ethical approval was given and informed consent for the use of (coded) images was waived by the Maastricht University Medical Centre ethical committee, as the data was analyzed anonymously in accordance with the Institutional Review Board guidelines (IRB no. 13-02-2015).

Radiotherapy was performed according to the current GEC-ESTRO guidelines; this consisted of external beam radiation therapy (EBRT) followed by MRI-guided brachytherapy (BCT). The first BCT was applied during the fifth week of radiotherapy, and the overall treatment time for radiotherapy was 6–7 weeks.

Patients were treated with either concurrent chemotherapy or hyperthermia (if concurrent chemotherapy was contraindicated or in case of FIGO 4B disease after neoadjuvant chemotherapy) according to local clinical guidelines.

### PET/MRI imaging protocol

A whole-body [^18^F]-FDG-PET/MRI was performed on a 3.0-Tesla Biograph mMR PET/MRI scanner (Siemens) with a 4.4-mm PET resolution (full width at half maximum).

Patients were fasting for at least 6 h before the examination; a blood glucose level (all < 10 mmol/L) was obtained in all patients. Body weight adapted intravenous administration of 2 Mbq/kg of 18 fluorodeoxyglucose was performed 45 min before the PET-MRI. The PET images were acquired in 5-min bed positions. Attenuation correction was performed with a dual-echo VIBE DIXON that separates water and fat with TE1/TE2 = 1.23 ms/2.46 ms, TR = 3.6 ms, left-right FOV = 500 mm, and anterior-posterior FOV = 300 mm. The PET acquisition was reconstructed using the Siemens HD reconstruction algorithm (3 iterations, 21 subsets, 4 mm Gauss, matrix size 300) and corrected for attenuation, scatter, randoms, dead-time, and radioactive decay. PET/MRI images were fused and analyzed using the dedicated DICOM software (Osirix MD, Geneva).

Quantification of FDG uptake was performed by assessing the standardized uptake value (SUV; measured activity concentration [Bq/ml]  ×  body weight [g]/injected activity [Bq]).

A total body (from skull-base to groin) 2D T2-weighted fast spin-echo image in two planes (sagittal and axial) was performed (TR 2150/ TE 138 ms, 33 ETL, 1NSA, (0.98 × 0.98) × 6.50 mm^3^).

Second, diagnostic pelvic MRI images were performed in three planes (sagittal, axial, and coronal), with the axial and coronal planes angled perpendicular and parallel to the cervical axis, respectively (2D T2-weighted (T2 W) fast spin-echo images (TR 4000/TE 102 ms, 25 ETL, 3 NSA, (0.98 × 0.98)× 3.00 mm^3^ voxel at 3.0 T). Furthermore, the protocol consisted of a T1 in the coronal plane (TR 700/TE 11 ms, 57 ETL, 1 NSA, (0.98 × 0.98) × 1.1 mm^3^ voxel at 3.0 T) and diffusion weighted images (TR 9000/TE 80 ms, 1 ETL, 3 NSA, (0.98 × 0.98) × 5.0 mm^3^ voxel at 3.0 T) with B-values (50, 400, and 800).

Patients did not receive bowel preparation, bladder catheterization, or anti-spasmodic agents.

### Additional scans

The pre-treatment MRI was performed according to the same protocol on the 3.0 Tesla Bioraph mMR PET-MR (Siemens Magnetom Avanto, Erlangen, Germany) or on a 1.5-Tesla MRI unit (Intera Achieva); Philips Healthcare, Best, The Netherlands (in 5 patients). The protocol used in the latter was a standard protocol for diagnostic pelvic MRI images comparable with the 3.0 Tesla PET-MR protocol consisting of T2W, T1W, and diffusion weighted images.

PET pre-treatment was performed according to the same protocol on the 3.0 Tesla PET-MR or in 5 patients on a PET-CT scanner Gemini TF TOF 64 (Philips Healthcare, Best, The Netherlands) according to a similar protocol as described for the PET-MR.

### Image evaluation

In The Netherlands, radiologic assessment is done by a radiologist and assessment of nuclear imaging by a nuclear physician. If combined imaging, like PET and CT, is used, the radiologist and nuclear physician evaluate their findings together and give their combined result to the clinician. The MRI images were retrospectively independently analyzed by a radiologist (FB) and the PET images by a nuclear physician (SdV) with experience in oncologic imaging blinded to all patient information and patient outcome. After the initial analysis, both readers evaluated the PET-MRI images together, they discussed their separate findings and came to consensus. ROC curves to determine area under the curve were performed.

The readers were asked to assess the presence of residual tumor and/or metastasis based on a “subjective” visual assessment of the images using the following confidence level scores: 0, definitely no residual tumor/metastasis; 1, probably no residual tumor/metastasis; 2, unclear; 3, probably residual tumor/metastasis; 4, definitely residual tumor/metastasis. The readers were not given any instructions or asked to search for certain criteria and were free to interpret the scans based on prior experience.

Second, at the consensus meeting, they scored diagnostic confidence. This was scored on a similar 5-point Likert scale: 0, certainly no increase in confidence; 1, probably no increase; 2, unclear; 3, probably increase; 4, definitely increase. Change in diagnostic confidence was calculated with a predefined cut-off (score 2); only scores 3 and 4 were seen as a change of diagnostic confidence.

All measurements were performed on a dedicated DICOM system (Osirix MD, Geneva).

### Standard of reference

Local residual tumor was defined as tumor at the original tumor site (cervix, vagina, parametria, bladder, or rectum).

The presence or absence of a local residual tumor was determined by:

1. Histopathology of the surgical resection specimen.
2. The results of a post-treatment gynecologic examination (under anesthesia, with or without biopsy) performed 3 months after completion of the entire radiation treatment and at least 12 months of documented follow-up. Follow-up consisted of three monthly check-up by alternating a gynecologic and radiation oncologist and if indicated with additional imaging and/or tumor markers conform clinical guidelines.

Regional residual tumor was defined as residual and/or recurrent lymphatic tumor within the irradiated volume (i.e., pelvic or lower para-aortic lymph nodes). Distant metastasis was defined as metastasis outside of the irradiated volume. Regional tumor and distant metastasis were proven either by histopathology or the detection of a growing tumor mass during subsequent imaging analysis.

After radiation therapy in case of isolated local residual disease without metastasis, patients were subjected to salvation surgery. If distant metastasis was present, palliative therapy was proposed. In case of no evidence of disease, follow-up was performed according to the clinical guidelines.

### Statistical analysis

Statistical analyses were performed using SPSS Statistics v20.0 (SPSS Inc., Chicago, Illinois) and Stata v11.0 (StataCorp LP, Texas).

Receiver operating characteristic (ROC) curves were constructed to evaluate diagnostic performance for (a) MRI, (b) PET, and (c) combined PET-MRI analysis. Corresponding areas under the ROC curve (AUC) were compared according to the method described by De Long et al. [13]. *P*-values less than 0.05 were considered statistically significant.

## Results

### Patient and treatment characteristics

Between July 2014 and October 2015, out of a prospective cohort of 11 patients, 10 patients obtained a PET-MRI. One patient did not have PET/MRI due to technical problems. Table 1 shows the baseline characteristics.

**Table 1:** Patient and tumor characteristics

Patient	Age (years)	Histological sub-type	FIGO	Diameter (cm)	Para-iliacal lymph nodes suspected on MR or PET-CT	Therapy
1	42	Squamous cell	IIB	5	**+**	CRT
2	51	Squamous cell	IIB	6	**–**	CRT
3	65	Adenosquamous	IIB	7	**+**	CRT
4	60	Squamous cell	IIB	4	**–**	CRT
5	45	Squamous cell	IIB	5	**–**	CRT
6	41	Squamous cell	IIB*	4	**+**	Neoadjuvant CHT + HT
7	50	Adenocarcinoma	IIB**	6	**+**	Neoadjuvant CHT + HT + RT on oligometastasis
8	30	Adenosquamous	IIB	5	**–**	CRT
9	53	Squamous cell	IVA	4	**–**	Neoadjuvant CHT + HT
10	27	Adenocarcinoma	IB2	4	**+**	CRT

*CRT* chemoradiation treatment, *HT* radiation treatment with hyperthermia

*MRI/PET/PA showed jugular metastasis

**MRI/PET/PA showed glenoid oligometastasis

All patients received treatment with curative intent. Two patients (patients 6 and 7) had distant metastasis (jugular lymph node and glenoid) on pretreatment MR/PET-CT, they received neoadjuvant chemotherapy (clinical stage IIB, histological stage IVB).

The median time interval between last treatment and the PET/MRI was 11 weeks (8–15 weeks).

### Diagnostic confidence, change of opinion, and change of policy

Table 2 shows the score for residual disease based on MRI, PET, and PET-MRI consensus. The reference standard, outcome, and additional treatments are shown. The functional MRI and PET parameters (ADC_mean_ and SUV_max_) after treatment are shown.

**Table 2:** Score for local residual tumor based on MRI (radiologist), PET (nuclear physician), and PET-MRI (consensus radiologist and nuclear physician) performed 3 months after treatment, quantitative measurements, reference standard, and outcome

Patient	MRI_post_ ADC_mean_	PET_post_ SUV_max_	MRI residual	PET residual	PET-MRI residual	Reference standard		Outcome
						Local disease	Distant metastasis	
1	1.5	1.8	– –	–	– –	No	No	FU: NED 20 months
2	1.4	2.8	–	–	– –	No	No	FU: NED 26 months
3	1.3	2.9	–	+/−	– –	No	No	FU: NED 25 months
4	1.3	3.0	+	+	+/−	No	No	FU: NED 27 months
5	0.7	3.2	– –	+	–	Yes*	Yes	Palliation CHT
6	1.3	3.6	+	+ +	+ +	Yes	Yes	Palliation CHT, diseased
7	1.6	3.0	+	– –	+/−	No	Yes	Multiple meta, palliative
8	2.0	2.6	+/−	–	+	No	No	Salvage surgery, PA: no tumor. NED 21 months
9	1.5	5.1	+/−	+ +	+	Yes	No	Salvage surgery, FU: liver metastasis 3 months after surgery; palliative CHT
10	1.6	7.3	–	+	– –	No	Yes	Palliative CHT

Corresponding symbols: − −, definitely no residual tumor; −, probably no residual tumor; +/−, unclear possibly residual tumor; +, probably residual tumor; + +, definitely residual tumor/metastasis

*Abbreviation*: *PA* pathology, *NED* no evidence of disease, *FU* follow-up, *CHT* chemotherapy

*No pathology of local residual disease, growing mass on subsequent scans

Tables 3 and 4 show the score for residual and distant disease together with the change of opinion and change of policy for both the MRI alone, PET alone, and both modalities combined.

**Table 3:** Local residual tumor based on MRI (radiologist), PET (nuclear physician), and PET-MRI (consensus radiologist and nuclear physician) scores and change of opinion and policy

Patient	MRI local	PET local	MRI-PET local	Change of opinion (reason)	Policy of change
1	TN	TN	TN	−	−
2	TN	TN	TN	−	−
3	TN	Equivocal	TN	**+** PET artifact	**+** Follow-up
4	FP	FP	Equivocal	**+** No PET uptake at suspicious MRI lesion	**+** Additional imaging
5	FN	TP	FN	**+** FDG uptake artifact at PET-MR	− Metastasis
6	TP	TP	TP	−	−
7	FP	TN	Equivocal	**+** Consensus + MRI, non-suspicious PET	+ Additional imaging
8	Equivocal	TN	FP	**+** Consensus PET-MR, PET hard to interpret due to low-FDG glucose uptake	**+** Salvage surgery
9	Equivocal	TP	TP	**+** MR partial volume, clear FDG uptake	**+** Salvage surgery
10	TN	FP	TN	**+** PET hotspot outside cervical tissue	− Metastasis

*Abbreviation*: *TN* true negative, *TP* true positive, *FP* false positive, *FN* false negative, + positive, − negative

**Table 4:** Metastases based on MRI (radiologist), PET (nuclear physician), PET-MRI (consensus radiologist and nuclear physician), and the reference standard, and change of opinion and policy

Patient	MRI metastases	PET metastases	MRI-PET metastases	Regional or distant disease	Change of opinion (reason)	Policy of change
1	TN	TN	TN	−	−	−
2	TN	TN	TN	−	−	−
3	TN	TN	TN	−	−	−
4	TN	Equivocal	Equivocal	− Primary lung tumor	**+** Primary lung tumor	**+** Additional therapy
5	TP	TP	TP	**+** Pelvic node	−	−
6	FN	TP	TP	**+** Multiple	**+** FDG-positive metastases	**+** Palliation
7	FN	FN	FN	**+** Multiple	− False negative	−
8	TN	TN	TN	−	−	−
9	TN	TN	TN	−	−	−
10	FN	TP	TP	**+** Para-aortic node	**+** FDG-positive metastases	**+** Palliation

*Abbreviation*: *TN* true negative, *TP* true positive, *FP* false positive, *FN* false negative + positive, − negative

Table 5 shows the diagnostic accuracy and diagnostic confidence.

**Table 5:** Diagnostic performance and confidence for MRI, PET and PET-MRI for assessing local residual cervical cancer

Modality	AUC (Standard error)	*P* value	Diagnostic confidence
MRI	0.55 (0.24)	0.008*	80%*
PET	0.95 (0.06)	0.65*	90%*
PET-MRI	0.83 (0.16)	^	^

*AUC* (area under ROC curve)

*Compared to PET-MRI

^No comparison

After radiation treatment, one patient had local residual tumor only; two patients had distant metastasis only and two had both local residual disease and distant metastasis. The remaining four patients had no evidence of disease after a median follow-up of 25 months (range 20–27 months) (Table 2). Two patients with suspected isolated local residual tumor underwent salvage surgery. In one patient (patient 8), salvage surgery was performed based on subsequent PET-MRI imaging and two biopsies showing possible minimal residual tumor. Pathologic examination of the uterus after salvage surgery however did not show active residual tumor; therefore, this patient was regarded as negative for local residual cancer. During the PET-MRI consensus meeting, the radiologist had a suspicion for local residual disease based on an isointense, nodular lesion in the cervix. The nuclear physician did not see any FDG hotspots; however, the scan was hard to interpret due to low-FDG uptake. Based on the MRI signs, they came to consensus and false positively decided that there probably was a residual tumor.

For MRI, the accuracy improved mainly due to increase in confidence of the observer. Patient 9 was scored doubtful on the initial MRI; however, the availability of PET resulted in a correct change of opinion to probably local residual tumor. Also, two MRIs were scored as probably residual disease; however, after the consensus meeting, these patients were downgraded to uncertain, therefore diminishing false positive results. Due to the small population, both shifts in scores result in a relatively large increase in AUC.

Patient 5 had a false negative score for residual local disease both on MRI and PET-MRI, but because of the presence of distant metastasis, this result had no therapeutic consequences. However, if metastases would not have been present, the PET result (probably residual disease) would have had clinical consequences. The local residual disease was shown by an increasing local mass with subsequent imaging.

The two patients (patients 6 and 10) with local residual disease and distant metastasis were correctly depicted by the PET component; MRI did not show these metastases.

Second, Tables 3 and 4 show the cases were a change of policy occurred due to the consensus meeting. A change of policy was shown, mainly due to the decrease of false negative results. For example, patient 9 based on MRI that would have been allocated to follow-up with additional imaging became the candidate for salvage surgery based on PET-MRI.

For patients 6 and 7, based on MRI, an isolated local disease was suspected, combination with PET revealed the presence of distant metastasis so that salvage surgery was omitted. On the contrary, patient 8 was incorrectly classificated as residual tumor by combined PET-MRI analyses, but pathology of the biopsies were inconclusive as well. For patient 4, PET and PET-MR imaging showed a second primary tumor, resulting in additional treatment for this patient.

### Diagnostic performance for assessment of response

**Fig. 1:**
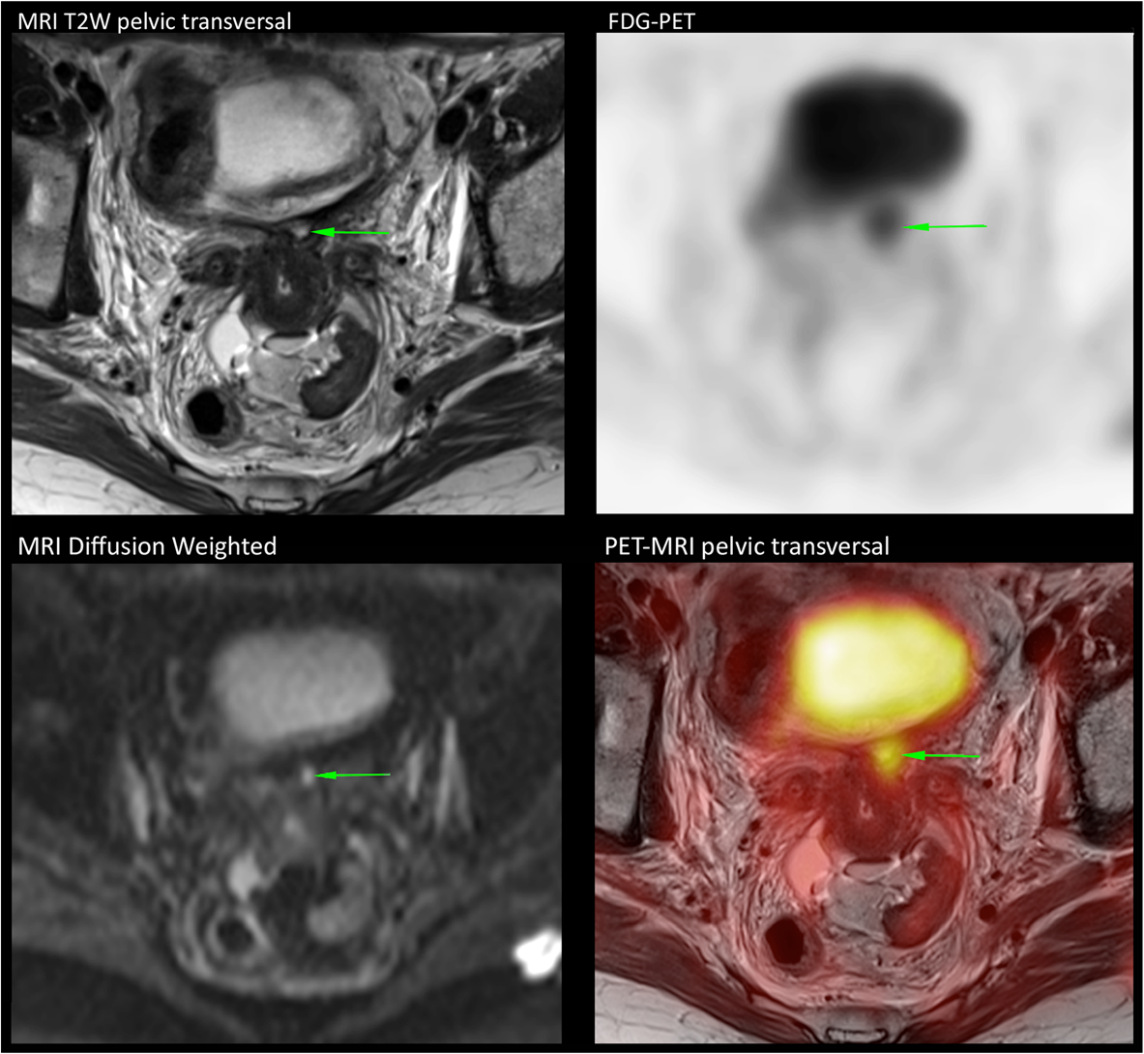
MRI T2W, diffusion-weighted MRI with ADC map, FDG-PET, and PET-MRI fusion showing local residual cancer (green arrow)

Figure 1 shows the T2W MRI, DW-MRI corresponding ADC map, FDG-PET, and the fusion images in a patient with local residual tumor after chemoradiation treatment. Additional Fig. 2 shows PET-MR images showing para-aortic lymph node metastasis.

**Fig. 2:**
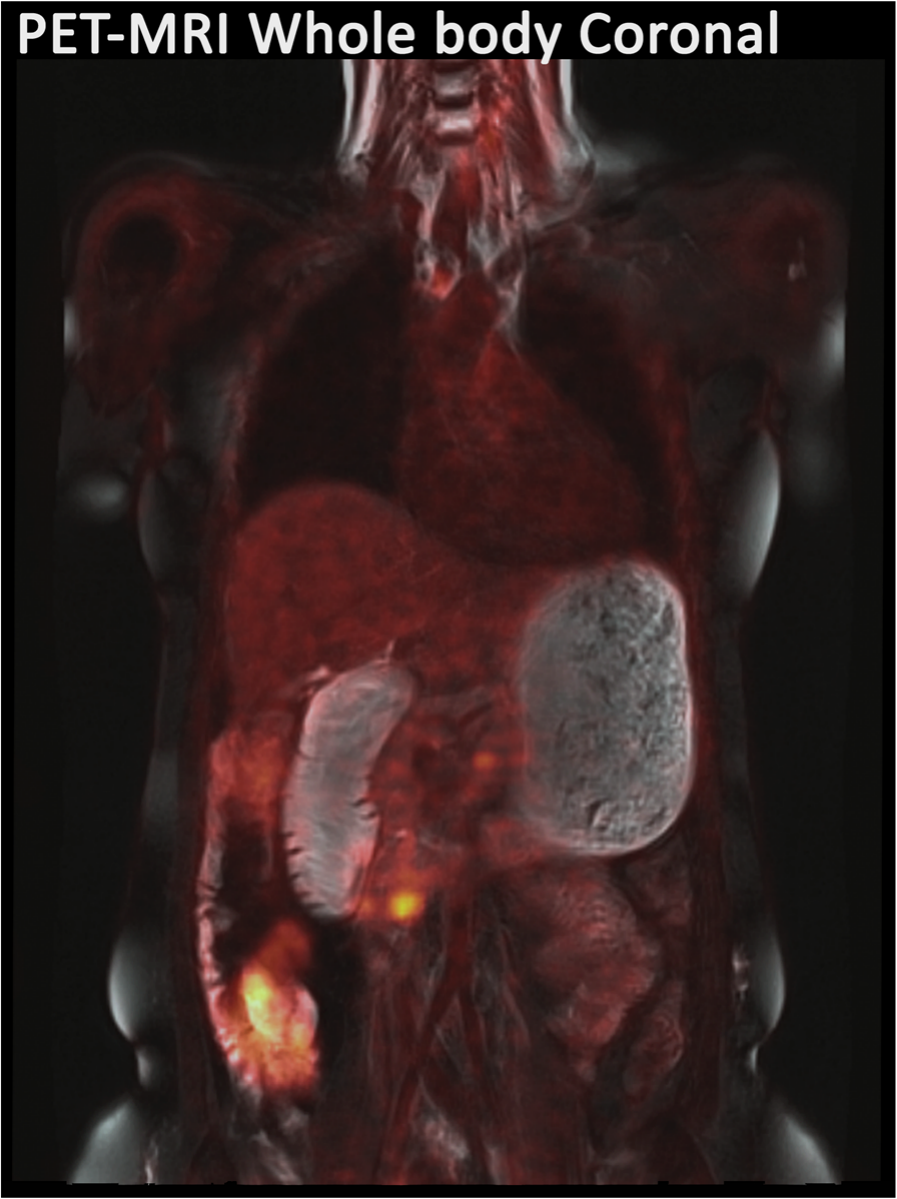
Whole-body PET-MRI fusion showing para-aortic lymph node metastasis

Table 5 shows the diagnostic performance of both readers based on their individual “subjective” visual response and there consensus “subjective” response.

Despite our small number of patients, PET-MRI evaluation showed a statistical higher AUC 0.83 versus 0.54 for MRI alone (*p* < 0.01). Compared to PET analyses, no statistical difference could be shown.

## Discussion

In this pilot study, we showed an increase in diagnostic confidence for both the radiologist and nuclear physician. Our results show that this increase in confidence is accompanied by an increase in diagnostic accuracy for assessing local residual cervical carcinoma after radiation treatment.

This is of major importance for the clinical setting, to make correct treatment decisions by the multi-disciplinary team.

The combined assessment of the PET-MRI resulted in a change of policy in 50% of patients.

Our hypothesis that the differentiation between active residual tumor and fibrotic tissue could be diminished by the additional PET data was partly correct as in 2 of 10 patients false positive MRI results could be corrected. We also showed a correct upstaging to probably residual disease with the PET data available, decreasing false negative results.

This is the first study assessing PET-MRI for evaluation of response 3 months after radiation treatment. Multiple research groups showed surveillance PET-CT 3 months after treatment to be predictive for tumor response; moreover, Siva et al. allocated patients with isolated local and nodal disease to salvage treatment with a high overall survival rate [5, 7].

MRI is also proposed as a surveillance scan 3 months after radiation treatment. However, response assessment is hampered by radiation-induced changes as fibrosis, necrosis, inflammation, and edema; false positive results up to 50% are common [9, 10]. These findings are supported by a recent study by Kim et al.; they showed 60% of patients with residual cervical cancer detected on post-radiation treatment MRI to remain disease free without disease progression [4]. Careful observation without salvage surgery was suggested as an option for patients with tumor < 2 cm on post-treatment MRI. These findings highlight the necessity for more accurate evaluation of MRI to diminish false positive results and to predict which suspected MRI lesions will progress over time.

Future studies will give more insight on the value of MRI and the timing of tumor regression after radiation treatment. Additional information about tumor biology could also diminish false positive results by differentiating between active residual cervical cancer and radiation-induced changes/non-active residual cancer in regression. Diffusion-weighted MRI showed some potential based on apparent diffusion coefficient (ADC); cut-off values remain unclear [14]. However, especially tumor glucose uptake as assessed by PET could prove to be of value; however, no studies performed both MRI and PET after radiation treatment to assess residual cervical cancer. Possible our false positive PET-MR result in one patient could be explained by a delayed tumor regression after radiation treatment.

Due to our small study population, we were not able to assess the quantitative functional data of the PET and MRI component. These apparent diffusion coefficient (ADC) and standard uptake value (SUV) are promising functional parameters in the course of radiation treatment. Due to the hybrid combination of PET and MRI, a reliable comparison between these two parameters becomes available.

In future trials, we will assess these parameters as these might be independent and subsequently both potentially increase diagnostic accuracy [15–17].

PET-MR has previously been used for suspected recurrence of gynecological malignancies including cervical carcinoma. A study performed by Gruneisen et al. showed PET-MR to be superior to whole-body MRI for detecting suspected recurrence while improving diagnostic confidence for the observers. This result is in concordance with our recent findings [11].

The same study group also evaluated PET/MRI versus PET/CT for suspected recurrence of gynecological malignancies. They showed comparable diagnostic accuracy for both methods. For diagnostic confidence of the readers, PET/MRI showed higher values for both malignant and benign lesions. Due to the small study populations with different gynecologic malignancies and different histological subtypes, these results have to be interpreted with caution [11, 12].

Our study has some limitations. A main limitation is our small patient population; therefore, conclusions have to be interpreted with caution. Despite our small population, promising results supporting the use of PET-MRI for evaluation of cervical carcinoma after radiation treatment could be shown. All patients allocated to radiation treatment by our team were consecutively scanned with the exception of one patient (no PET-MRI performed); therefore, the risk of selection bias is rather low.

Second, due to the nature of treatment and follow-up, it is not possible to have pathology as the golden standard in patients with a complete response. But we believe that we were able to provide sufficient follow-up, making false negative results rather unlikely.

## Conclusions

In conclusion, a combined PET-MR evaluation shows promising results for assessing treatment response after radiation treatment for cervical cancer, especially increasing diagnostic confidence of the observers.

This report shows the benefit of PET addition to MRI as both false positive and false negative MRI findings could be diminished by the addition of the PET.

## References

[1] Sardain H, Lavoue V, Redpath M, Bertheuil N, Foucher F, Leveque J (2015). Curative pelvic exenteration for recurrent cervical carcinoma in the era of concurrent chemotherapy and radiation therapy. A systematic review. Eur J Surg Oncol.

[2] Kim TH, Kim MH, Kim BJ, Park SI, Ryu SY, Cho CK (2017). Prognostic importance of the site of recurrence in patients with metastatic recurrent cervical cancer. Int J Radiat Oncol Biol Phys.

[3] Koh WJ, Greer BE, Abu-Rustum NR, Apte SM, Campos SM, Chan J (2013). Cervical cancer. J Natl Compr Cancer Netw.

[4] Kim JY, Byun SJ, Kim YS, Nam JH (2017). Disease courses in patients with residual tumor following concurrent chemoradiotherapy for locally advanced cervical cancer. Gynecol Oncol.

[5] Schwarz JK, Siegel BA, Dehdashti F, Grigsby PW (2007). Association of posttherapy positron emission tomography with tumor response and survival in cervical carcinoma. JAMA.

[6] Choi J, Kim HJ, Jeong YH, Lee JH, Cho A, Yun M (2014). The role of (18) F-FDG PET/CT in assessing therapy response in cervix cancer after concurrent chemoradiation therapy. Nucl Med Mol Imaging.

[7] Siva S, Deb S, Young RJ, Hicks RJ, Callahan J, Bressel M (2015). (1)(8)F-FDG PET/CT following chemoradiation of uterine cervix cancer provides powerful prognostic stratification independent of HPV status: a prospective cohort of 105 women with mature survival data. Eur J Nucl Med Mol Imaging.

[8] Lee SI, Catalano OA, Dehdashti F (2015). Evaluation of gynecologic cancer with MR imaging, 18F-FDG PET/CT, and PET/MR imaging. J Nucl Med.

[9] Mongula J, Slangen B, Lambregts D, Bakers F, Mahesh S, Lutgens L (2016). Predictive criteria for MRI-based evaluation of response both during and after radiotherapy for cervical cancer. J Contemp Brachytherapy.

[10] Vincens E, Balleyguier C, Rey A, Uzan C, Zareski E, Gouy S (2008). Accuracy of magnetic resonance imaging in predicting residual disease in patients treated for stage IB2/II cervical carcinoma with chemoradiation therapy: correlation of radiologic findings with surgicopathologic results. Cancer.

[11] Grueneisen J, Beiderwellen K, Heusch P, Gratz M, Schulze-Hagen A, Heubner M (2014). Simultaneous positron emission tomography/magnetic resonance imaging for whole-body staging in patients with recurrent gynecological malignancies of the pelvis: a comparison to whole-body magnetic resonance imaging alone. Investig Radiol.

[12] Beiderwellen K, Grueneisen J, Ruhlmann V, Buderath P, Aktas B, Heusch P (2015). [(18)F]FDG PET/MRI vs. PET/CT for whole-body staging in patients with recurrent malignancies of the female pelvis: initial results. Eur J Nucl Med Mol Imaging.

[13] DeLong ER, DeLong DM, Clarke-Pearson DL (1988). Comparing the areas under two or more correlated receiver operating characteristic curves: a nonparametric approach. Biometrics.

[14] Levy A, Caramella C, Chargari C, Medjhoul A, Rey A, Zareski E (2011). Accuracy of diffusion-weighted echo-planar MR imaging and ADC mapping in the evaluation of residual cervical carcinoma after radiation therapy. Gynecol Oncol.

[15] Grueneisen J, Beiderwellen K, Heusch P, Buderath P, Aktas B, Gratz M (2014). Correlation of standardized uptake value and apparent diffusion coefficient in integrated whole-body PET/MRI of primary and recurrent cervical cancer. PLoS One.

[16] Brandmaier P, Purz S, Bremicker K, Hockel M, Barthel H, Kluge R (2015). Simultaneous [18F]FDG-PET/MRI: correlation of apparent diffusion coefficient (ADC) and standardized uptake value (SUV) in primary and recurrent cervical cancer. PLoS One.

[17] Ippolito D, Fior D, Trattenero C, Ponti ED, Drago S, Guerra L (2015). Combined value of apparent diffusion coefficient-standardized uptake value max in evaluation of post-treated locally advanced rectal cancer. World J Radiol.

